# Targeting macrophages to treat intracranial aneurysm

**DOI:** 10.18632/oncotarget.21368

**Published:** 2017-09-28

**Authors:** Tomohiro Aoki, Rie Yamamoto, Shuh Narumiya

**Affiliations:** Department of Molecular Pharmacology, Research Institute, National Cerebral and Cardiovascular Center, Suita, Osaka, Japan; Alliance Laboratory for Advanced Medical Research, Medical Innovation Center, Kyoto University Graduate School of Medicine, Sakyo-ku, Kyoto, Kyoto, Japan

**Keywords:** intracranial aneurysm, macrophage, prostaglandin, EP2, S1P1

Although the recent advancement in medical care and modalities have greatly improved outcome of various diseases, there are still ones to which treatment intervention is still unsatisfactory. One of representative example of such a disease is subarachnoid hemorrhage due to rupture of an intracranial aneurysm (IA). Subarachnoid hemorrhage has quite a poor outcome, i.e. mortality rate of 50 %. Moreover, the prevalence of IAs in general public is high about 1 to 5 %. Thus, a pre-emptive treatment of IAs to prevent rupture is mandatory for social health. However, currently, there is no medical therapy available for IAs. Thereby, it is socially demanded to develop a novel therapeutic drug for IA treatment based on its pathogenesis.

**Figure 1:**
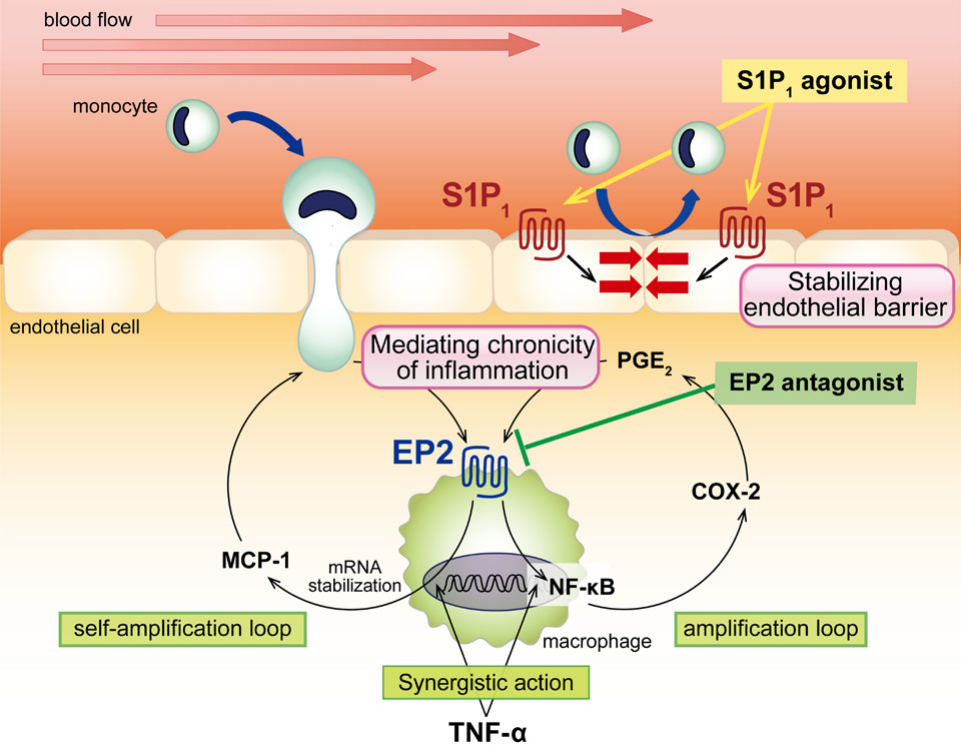
Schematic presentation of the role of EP2 and S1P_1_ signaling in the activation or recruitment of macrophages and potential of an EP2 antagonist or a S1P_1_ agonist as a therapeutic drug for intracranial aneurysm.

Recent experimental findings have revealed some principal mechanisms underlying the disease progression and proposed potential therapeutic targets, making development of effective medical therapy more likely. Especially, in a series of studies, the crucial contribution of a long-lasting ‘chronic’ inflammation to the pathogenesis of IAs has been demonstrated and thus nowadays IA is considered as a chronic inflammatory disease affecting intracranial arteries. Notably, we and others have revealed involvement of macrophages, which are the most abundant inflammatory cells detected in IA lesions of both human and animal models [1, 2], to progression of IAs through regulating inflammatory responses in situ [3, 4]. For example, the pharmacological depletion of macrophages [3] and genetic deletion of a chemoattractant for macrophages [3, 4], MCP-1, both significantly suppress inflammatory responses in lesions and IA. Thus, a macrophage itself, factors mediating macrophage infiltration or ones functioning in macrophage to evoke inflammation can become a therapeutic target for IAs. In addition, such a factor may be applicable to develop drugs for many other macrophage-related diseases. As described in greater detail below, we have recently identified Sphingosine-1-phosphate receptor type 1 (S1P_1_) and PGE receptor subtype 2 (EP2) as a strong therapeutic target [5, 6] (Figure 1).

Recently, we have identified S1P_1_ as a factor related with trans-endothelial migration of macrophages in IA lesions and proposed the potential of a selective S1P_1_ agonist, ASP4058, as a candidate for treatment [6] (Figure 1). This receptor is expressed in endothelial cells of arterial walls including IA lesions. ASP4058 acts on endothelial cells as an agonist to reduce permeability and migration of macrophages across endothelial monolayer in *in vitro* system [6]. In IA lesions of rats, a part of endothelial cell junctions are dissociated and vascular permeability is increased, which can be restored by administration of ASP4058 [6]. Presumably as a result of stabilization of endothelial barrier, oral administration of ASP4058 significantly suppresses macrophage infiltration and IA progression [6]. Importantly, a non-selective S1P receptor agonist, fingolimod, fails to suppress but rather exacerbates IA progression [6], suggesting the presence of a S1P receptor oppositely functioning from S1P_1_ and thus the importance of a prominent selectivity to S1P_1_ over other S1P receptor subtypes. As clinical trials including Phase III studies for some selective S1P_1_ agonists are ongoing for multiple sclerosis or other autoimmune diseases, this class of drugs may be a leading candidate for clinical usage to treat IAs.

Another potential therapeutic target is a factor mediating an inflammatory response functioning in macrophages. We have currently identified the Prostaglandin E_2_ (PGE_2_)-EP2-NF-κB signaling cascade present in macrophages as a factor regulating such a chronic inflammation involved in the pathogenesis of IAs (Figure 1). PGE_2_ is the metabolite of arachidonic acid by the sequential enzymatic actions including cyclooxygenase (COX). Because the inhibitors of COX, NSAIDs (non-steroidal anti-inflammatory drugs), effectively suppress symptoms of acute inflammation (pain, fever, etc.), this PG cascade is considered as a major mediator of acute inflammation. In addition to such a traditional concept, recent experimental findings including ours have suggested the crucial role of PG cascade in formation and exacerbation of chronic inflammation promoting the IA pathogenesis [5, 7]. In brief, NF-κB activation, a hallmark of inflammation in IA lesions [8], can be detected in infiltrating macrophages and endothelial cells in IA lesions of mouse model at the early stage and, later on, spreads to entire arterial walls. Genetic deletion of EP2 or inhibition of NF-κB activation specifically in macrophages similarly suppresses IA formation, macrophage infiltration and expression of pro-inflammatory factors in lesions. Importantly, macrophage-specific deletion of EP2 results in suppression of NF-κB activation in entire IA lesions, suggesting the role of EP2 signaling in macrophages in maintenance of chronic inflammation in whole lesions. In human IA specimens, the presence and extent of EP2 expression is positively correlated with macrophage infiltration in lesions, confirming the clinical relevance of above studies. Intriguingly, PGE_2_-EP2 cascade mediates chronic inflammation at multiple steps. First, EP2 signaling synergizes with TNF-α present in microenvironment and amplifies cytokine-induced expression of pro-inflammatory genes to promote inflammatory responses. As COX-2, an inducible form of COX, becomes a representative example of such genes, the amplification-loop involving PGE_2_-EP2 cascade is formed making EP2-dependent inflammatory response exacerbated and long-lasting once after PGE_2_ is produced in response to inflammatory stimuli. Second, EP2 signaling enhances TNF-α-induced MCP-1 expression through stabilization of mRNA. Thereby, the self-amplification loop among macrophages is formed under EP2 signaling once after a small number of macrophages is recruited. Here noted that EP2 is induced at the transcription level in IA lesions, meaning the formation of EP2-dependent inflammatory microenvironment specifically in lesions. Thus, EP2 signaling becomes a major mediator of chronic inflammation contributing to the pathogenesis, making EP2 a strong and safer candidate of drugs for IA treatment.
